# Impfverhalten von Klinikpersonal verstehen – Ergebnisse der OKaPII-Studie 2023

**DOI:** 10.1007/s00103-024-03982-7

**Published:** 2024-12-03

**Authors:** Elisa Wulkotte, Nora Katharina Schmid-Küpke

**Affiliations:** Fachgebiet Impfprävention/STIKO, Abteilung für Infektionsepidemiologie, Robert Koch-Institut, Seestraße 10, 13353 Berlin, Deutschland

**Keywords:** Influenza-Impfung, Gesundheitspersonal, Impfverhalten, Behaviorale und soziale Faktoren, 5C-Modell, Influenza vaccination, Healthcare workers, Vaccination behavior, Behavioral and social determinants, 5C model

## Abstract

**Hintergrund:**

Das Potenzial der Influenza-Impfung zur Verhinderung der Erkrankung und Weiterverbreitung des Virus wird im klinischen Setting nicht ausgeschöpft. Ein Verständnis von Impfverhalten ist notwendig, um wirksame Maßnahmen zur Steigerung der Impfquote ergreifen zu können.

**Methodik:**

OKaPII ist eine jährliche, deutschlandweite Onlinebefragung von Klinikpersonal zur Influenza-Impfung. Es wurden Unterschiede im Impfverhalten nach Alter, Geschlecht und Beruf sowie im Wissen zwischen Ärzteschaft und Pflege getestet. Zusammenhänge zwischen psychologischen Determinanten und Impfverhalten wurden bei Ärzteschaft und Pflege mittels Regressionsanalysen identifiziert.

**Ergebnisse:**

An der Befragung (17.04.–15.05.2023) nahmen 15.312 Mitarbeitende aus 115 Kliniken teil. In der Saison 2022/2023 waren 58,7 % des befragten Klinikpersonals gegen Influenza geimpft (Pflege: 51,1 %; Ärzteschaft: 80,7 %). Die Impfentscheidung von Ärzteschaft und Pflegepersonal hing u. a. signifikant mit der Wahrnehmung der Impfung als gemeinschaftliche Maßnahme (Pflege: OR = 1,94; Ärzteschaft: OR = 1,89) sowie dem Vertrauen in die Sicherheit der Impfung (Pflege: OR = 1,90; Ärzteschaft: OR = 1,78) zusammen. Von den Wissensitems wurden 87,2 % von der Ärzteschaft und 62 % von der Pflege richtig beantwortet.

**Diskussion:**

Es bestehen deutliche Unterschiede in den Impfquoten zwischen Berufsgruppen in deutschen Kliniken. Impflücken bestehen seit Jahren, insb. bei Pflegekräften. Zielgruppengerechte Interventionen sollten den Schutzgedanken vulnerabler Personen und das Vertrauen in die Sicherheit der Impfung fördern. Verstärkte Aufklärung, v. a. zu Sicherheitsfragen der Impfung, kann die Impfentscheidung positiv beeinflussen. Es sollten Möglichkeiten geschaffen werden, die Impfung trotz Zeitnot am Arbeitsplatz wahrzunehmen.

**Zusatzmaterial online:**

Zusätzliche Informationen sind in der Online-Version dieses Artikels (10.1007/s00103-024-03982-7) enthalten.

## Abstract (en)

**Background:**

Influenza vaccination coverage in Germany is generally low. Even in hospitals, the demand for influenza vaccination is lacking although it has the potential to prevent the spread of the influenza virus and diseases. To take effective actions, a deeper understanding of vaccination behavior of healthcare workers is crucial.

**Methods:**

OKaPII is an annual German-wide online survey of clinical staff on influenza vaccination. Differences in vaccination behavior were tested for occupation, sex, and age, and differences in knowledge were tested between physicians and nurses. We used logistic regressions to analyze associations between psychological determinants and vaccination behavior among physicians and nursing staff.

**Results:**

From 115 clinics, 15,312 employees participated in the survey (17 April to 15 May 2023). In the 2022/23 season, 58.7% of the participants were vaccinated against influenza. The vaccination coverage was 80.7% for physicians and 51.1% for nursing staff . The vaccination behavior of physicians and nursing staff was significantly associated with collective responsibility and confidence in vaccination safety. Of the knowledge items, 87.2% were answered correctly by physicians and 62% by nursing staff.

**Discussion:**

We identified significant differences in vaccine uptake between occupational groups in German hospitals. Low vaccination coverage has existed for years, especially among nursing staff. Tailored interventions should promote the idea of protecting vulnerable people and confidence in the safety of vaccination. Increased education, especially on vaccination safety issues, can have a positive impact on the decision to vaccinate. Possibilities to get vaccinated despite time constraints in the workplace should be provided.

## Hintergrund

Influenza gehört in Deutschland zu den häufigsten Infektionskrankheiten: In den Saisons von 2017 bis 2020 waren zwischen 21 % und 28 % aller Meldungen an das Robert Koch-Institut hospitalisierte Influenza-Fälle [1–4]. Die Impfung gegen Influenza gehört neben allgemeinen Hygienemaßnahmen zu den wichtigsten Maßnahmen der Prävention. Die Ständige Impfkommission (STIKO) empfiehlt die saisonale Influenza-Impfung im Herbst für Personen mit einem erhöhten Risiko für einen schweren Krankheitsverlauf, für Schwangere und für Personen, die aufgrund ihres Berufs einem höheren Infektionsrisiko ausgesetzt sind [5]. Dabei ist Personal in medizinischen Einrichtungen eine wichtige Zielgruppe. Die Impfung bietet ihnen einen individuellen Schutz und kann die Transmission des Influenza-Virus in Einrichtungen mit vulnerablen Patient:innen reduzieren [6, 7]. In den Herbst- und Wintermonaten sehen sich Kliniken verstärkt mit Personalengpässen – insbesondere in der Pflege [8] – und teils folgenschweren nosokomialen Influenza-Infektionen bei Patient:innen konfrontiert [9, 10]. Diese Problematik wurde während der COVID-19-Pandemie verstärkt und wird auch zukünftig eine Herausforderung bleiben [11, 12]. Eine hohe Influenza-Impfquote bei medizinischem Personal hat das Potenzial, diese Herausforderungen zu reduzieren und die Mitarbeitenden selbst zu schützen [13, 14].

Vergangene Erhebungen weisen jedoch seit Jahren auf teils große Impflücken bei Personal in deutschen Kliniken hin [15–17]. Vor der Pandemie berichteten 79,3 % der Ärzteschaft und 46,7 % des Pflegepersonals, dass sie sich in der Saison 2019/2020 haben impfen lassen. Die durchschnittliche Impfquote bei Klinikpersonal lag auch in den Jahren zuvor nie über 60 % [15]. Um das Präventionspotenzial der Impfung besser auszuschöpfen, ist es notwendig, die behavioralen und sozialen Faktoren von Gesundheitsverhalten zu verstehen. Nur so können effektive Maßnahmen zur Steigerung der Impfquoten für definierte Zielgruppen getroffen werden [18]. Die Relevanz von Verhaltens- und Sozialwissenschaften bei Public-Health-Fragestellungen wurde einmal mehr durch die COVID-19-Pandemie bewiesen [19, 20].

Das Robert Koch-Institut führt seit 2016 die „**O**nline-Befragung von **K**r**a**nkenhaus-**P**ersonal zur **I**nfluenza-**I**mpfung“ (OKaPII) durch. OKaPII ermöglicht ein jährliches, deutschlandweites Monitoring der Influenza-Impfung bei Klinikpersonal. Dabei wird nicht nur das Impfverhalten erfasst, sondern auch dessen Determinanten. Darüber hinaus erhalten teilnehmende Kliniken eigene Ergebnisberichte, die bei der Vorbereitung von klinikinternen Impfaktivitäten für die kommende Influenza-Saison unterstützen können.

Ziel der vorliegenden Analysen ist es, das Influenza-Impfverhalten von Klinikpersonal in der Saison 2022/2023 zu verstehen, um Ansatzpunkte für Maßnahmen zur Steigerung der Impfquoten identifizieren zu können. Dafür sollen folgende Fragestellungen beantwortet werden:

- Wie hoch waren die Influenza-Impfquoten in den befragten Berufsgruppen in der Saison 2022/2023?
- Welche psychologischen Determinanten bestimmen das Impfverhalten von Ärzt:innen und Pfleger:innen?
- Wie ist der Wissensstand bei der Ärzteschaft und dem Pflegepersonal zur Influenza-Impfung und zur Erkrankung?

## Methodik

Die OKaPII-Studie ist eine jährliche Onlinebefragung von Klinikpersonal. Es handelt sich um ein multizentrisches Design, d. h., es wurden Kliniken rekrutiert, die wiederum ihre Mitarbeitenden zur Studienteilnahme aufgerufen haben. Die Rekrutierung der Kliniken erfolgte über Aufrufe per E‑Mail und in der Zeitschrift *Das Krankenhaus*. Teilnehmende Klinikmitarbeiter:innen konnten sich nach der Befragung an einem Gewinnspiel beteiligen. Weitere Informationen zum Studiendesign wurden an anderer Stelle veröffentlicht [21, 22].

Eine Übersicht zu den Variablen des Fragebogens ist im Onlinematerial dargestellt (Tabelle A1). Zu Beginn wurden Angaben zur Soziodemografie erfragt, darunter Geschlecht, Alter (in Jahren) und Berufsgruppe. Die Teilnehmenden gaben anschließend an, ob sie sich in der vergangenen Saison 2022/2023 gegen Influenza haben impfen lassen (Impfstatus: 0 – ungeimpft, 1 – geimpft).

Um das Impfverhalten der Befragten erklären zu können, wurde die Kurzskala des 5C-Modells nach Betsch et. al. (2018; [23]) verwendet. Das Modell erfasst die psychologischen Determinanten der Inanspruchnahme von Impfungen. Dazu gehören *Confidence* (Vertrauen in Sicherheit und Wirksamkeit von Impfungen), *Collective Responsibility* (Wahrnehmung von Impfungen als gesellschaftliche Maßnahme), *Constraints* (Wahrnehmung von physischen Barrieren der Inanspruchnahme), *Calculation* (Ausmaß der Informationssuche) und *Complacency* (Risikowahrnehmung). Die Items des 5C-Modells wurden an die Influenza-Impfung angepasst. Die Kurzskala (5 Items) des 5C-Modells wurde um ein weiteres *Confidence*-Item aus der Langskala des Modells ergänzt, um sowohl das Vertrauen in die Sicherheit als auch in die Wirksamkeit der Influenza-Impfung zu erfassen. Beide Items wurden getrennt voneinander analysiert. So wurden die jährlich schwankende Wirksamkeit sowie der Umstand, dass die Wirksamkeit für die aktuelle Saison bei der Impfentscheidung noch nicht bekannt ist, als spezielle Eigenschaft der Influenza-Impfung berücksichtigt, die das Vertrauen in die Impfung beeinflussen kann. Die Befragten gaben auf einer 5‑Punkt-Likert-Skala an, inwiefern sie den Aussagen der Items zustimmen (1 – *stimme überhaupt nicht zu* bis 5 – *stimme voll und ganz zu*).

Darüber hinaus enthielt der Fragebogen in Summe 7 falsche und richtige Aussagen über die Influenza-Erkrankung und -Impfung (Wissensitems). Die Befragten haben angegeben, ob sie die Aussagen für richtig oder falsch halten oder ob sie es nicht wissen. Für beide Berufsgruppen wurden prozentuale Anteile pro Antwortkategorie angegeben (richtige Antwort, falsche Antwort, unsichere Antwort). Darüber hinaus wurde ein Wissensscore für die Berufsgruppen Ärzteschaft und Pflege berechnet. Dafür wurden die Antworten der Wissensitems umcodiert (1 – richtige Antwort, 0 – falsche oder unsichere Antwort) und anschließend addiert.

Die Analysen wurden mit dem Statistikprogramm R durchgeführt. Das dazugehörige Skript^1^ ist online bereitgestellt. Um die Influenza-Impfquote des befragten Klinikpersonals darlegen zu können, wurden Mittelwerte und dazugehörige 95 %-Konfidenzintervalle (KI) aus dem binären Item „Impfstatus“ berechnet. Die Impfquoten wurden stratifiziert nach den Merkmalen Geschlecht, Alter und Berufsgruppe dargestellt. Mithilfe von Chi^2^-Tests wurden Gruppenunterschiede in der Impfquote zwischen den Geschlechts‑, Alters- und Berufsgruppen getestet. Die psychologischen Determinanten wurden jeweils für die befragte Ärzteschaft und das befragte Pflegepersonal analysiert. Es wurden binär logistische Regressionsmodelle gerechnet, um zu prüfen, inwiefern die psychologischen Determinanten mit dem Impfstatus zusammenhängen. Die Zusammenhänge wurden für die Merkmale Geschlecht und Alter der befragten Person kontrolliert. Die Modellgüte wurde anhand des Akaike-Kriteriums (AIC) überprüft. Als statistisches Zusammenhangsmaß wurden Odds Ratios (OR) ausgegeben. Auch die Wissensitems wurden für die Ärzteschaft und das Pflegepersonal getrennt betrachtet. Prozentuale Angaben der einzelnen Antwortkategorien wurden deskriptiv dargestellt. Gruppenunterschiede im Wissensscore wurden mit einem T‑Test überprüft.

## Ergebnisse

An der OKaPII-Befragung vom 17.04. bis zum 15.05.2023 nahmen insgesamt 15.312 Mitarbeitende aus 115 Kliniken teil. Das entspricht 6,1 % aller Krankenhäuser in Deutschland [24]. Aufgrund der Rekrutierungsstrategie der Kliniken über einen Teilnahmeaufruf in einer Zeitschrift kann die Response-Rate der Kliniken nicht berechnet werden. Die Merkmale der teilnehmenden Kliniken sind im Onlinematerial dargestellt (Tabelle A2). Die Response-Rate der Mitarbeitenden in den Kliniken betrug 8,4 % (Anteil der Teilnehmenden unter allen Mitarbeitenden der teilnehmenden Kliniken). Tab. 1 zeigt die Charakteristika der Stichprobe. Das durchschnittliche Alter der Befragten lag bei 43 Jahren (SD = 12,3) und 72,3 % der Teilnehmenden waren weiblich. Die Berufsgruppe der Pflegekräfte war mit 31,3 % am stärksten in der Stichprobe vertreten und 15,8 % der Befragten waren im ärztlichen Dienst tätig.

**Tab. 1:** Beschreibung der Studienpopulation

	Absolute Häufigkeit (*N*)	Relative Häufigkeit (%)
*Alter in Jahren*		
18–29	3008	19,6
30–39	3673	24,0
40–49	3520	23,0
50–59	3917	25,6
≥ 60	1194	7,8
*Geschlecht*		
Weiblich	11.068	72,3
Männlich	4189	27,4
Divers	55	< 1
*Berufsgruppe*		
Pflegepersonal	4798	31,3
Verwaltung	3237	21,1
Ärzteschaft	2415	15,8
Sonstige	2196	14,3
Medizinisch-technisches Personal	969	6,3
Therapeutische Berufe	890	5,8
Laborpersonal	708	4,6
Reinigung, Küche und Hauswirtschaft	99	< 1
*Bundesland der Klinik*		
Nordrhein-Westfalen	3624	23,7
Niedersachsen	2242	14,6
Schleswig-Holstein	1815	11,9
Rheinland-Pfalz	1710	11,2
Baden-Württemberg	1525	10
Bayern	1523	9,9
Sachsen	1268	8,3
Hessen	395	2,6
Brandenburg	379	2,5
Bremen	244	1,6
Hamburg	190	1,2
Mecklenburg-Vorpommern	197	1,3
Sachsen-Anhalt	115	0,8
Thüringen	54	0,4
Berlin	39	0,3
Saarland	0	0

**Abb. 1:**
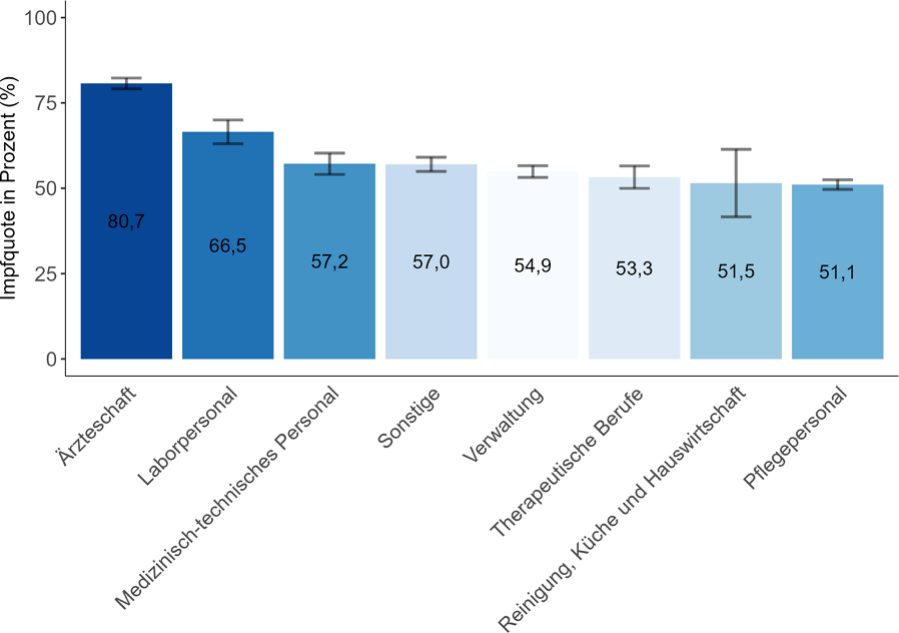
Influenza-Impfquote, inkl. 95 %-Konfidenzintervall, nach Berufsgruppe in der Saison 2022/2023 [39]

Die Impfquote für alle befragten Klinikmitarbeiter:innen lag bei 58,7 % (KI: 57,9–59,4). Männer haben die Influenza-Impfung mit 63,8 % signifikant häufiger in Anspruch genommen als Frauen (56,8 %; *chi*^*2*^(2) = 68,89; *p* < 0,001). Die Influenza-Impfquote unterschied sich signifikant zwischen den Altersgruppen (*chi*^*2*^(4) = 906,2; *p* < 0,001). Von den 18- bis 29-Jährigen haben sich 41 % in der Saison 2022/2023 gegen Influenza impfen lassen, in der Altersgruppe 30–39 Jahre waren es 51,8 % und bei den 40- bis 49-Jährigen 61,6 %. Die Impfquote für die Altersgruppe 50–59 Jahre lag bei 68,8 % und bei den ab 60-Jährigen waren 82,1 % geimpft. Darüber hinaus gab es signifikante Unterschiede bei der Inanspruchnahme der Influenza-Impfung zwischen den Berufsgruppen (*chi*^*2*^(7) = 650,51; *p* < 0,001, Abb. 1). Ärzt:innen nahmen die Influenza-Impfung in der Saison 2022/2023 signifikant häufiger in Anspruch als Pfleger:innen (*chi*^*2*^(1) = 591,2; *p* < 0,001).

**Abb. 2:**
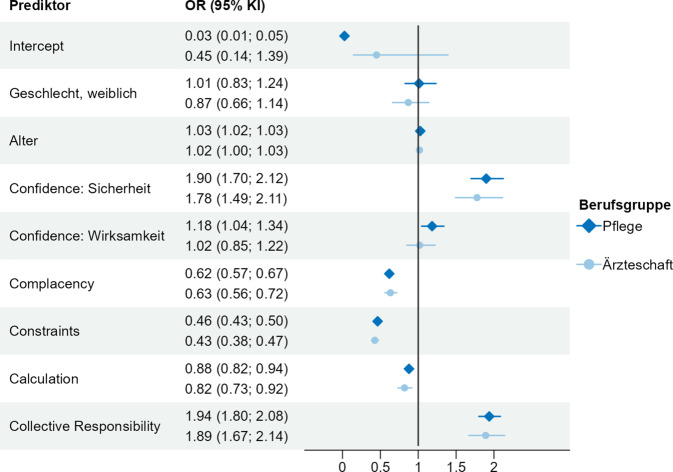
Zusammenhänge zwischen psychologischen Determinanten und Impfverhalten bei Pflegepersonal und Ärzteschaft – Ergebnisse der binärlogistischen Regressionsanalyse (*OR* Odds Ratios, *KI* Konfidenzintervall, *AIC* Akaike-Informationskriterium). Odds Ratios kleiner gleich 1 bedeuten einen negativen Zusammenhang mit Impfverhalten (geringere Chance, geimpft zu sein). Odds Ratios größer gleich 1 bedeuten einen positiven Zusammenhang mit Impfverhalten (höhere Chance, geimpft zu sein). *Quelle*: eigene Abbildung. AICPflege = 3388,4 (Referenz Nullmodell: 6627,6). AICÄrzteschaft = 1444 (Referenz Nullmodell: 2353,3)

Die Ergebnisse der binärlogistischen Regressionsmodelle für die Ärzteschaft und das Pflegepersonal zeigen, welche Determinanten mit dem Impfverhalten assoziiert waren (Abb. 2). Das Modell der Ärzteschaft basiert auf einer Stichprobe von *N* = 1946 geimpften Ärzt:innen und *N* = 461 ungeimpften Ärzt:innen. Die Voraussetzungen für eine binärlogistische Regression wurden geprüft und durch das Modell erfüllt (Unabhängigkeit der Variablen, keine Multikollinearität, Linearität des Logits). Bei der Ärzteschaft hingen alle untersuchten psychologischen Determinanten signifikant mit dem Impfverhalten zusammen, mit Ausnahme des Vertrauens in die Wirksamkeit der Impfung (OR = 1,02; 95 % KI: 0,85–1,22). Geschlecht und Alter waren nicht signifikant mit dem Impfverhalten der Ärzteschaft assoziiert. Die Odds Ratios zum Vertrauen in die Sicherheit der Influenza-Impfung (*Confidence*) und zur Wahrnehmung der Impfung als gesellschaftliche Maßnahme (*Collective Responsibility)* implizieren innerhalb des Modells die stärkste Assoziation mit Impfverhalten. Mit jedem Skalenpunkt zunehmender Zustimmung, dass die Impfung eine Maßnahme zum Schutz von Menschen mit schwachem Immunsystem ist, erhöhte sich die Chance der Ärzt:innen, geimpft zu sein, um den Faktor 1,89. Mit jedem Skalenpunkt zunehmender Zustimmung, dass vollstes Vertrauen in die Sicherheit der Influenza-Impfung besteht, erhöhte sich die Chance der Ärzt:innen geimpft zu sein um den Faktor 1,78. Abb. 2 zeigt weiterhin, dass *Constraints* (Alltagsstress als wahrgenommene Barriere), *Complacency* (geringe Risikowahrnehmung) und *Calculation* (hohes Ausmaß an Informationssuche) negativ mit dem Impfverhalten der Ärzteschaft assoziiert waren.

Auch für das Regressionsmodell der Pflege sind die oben genannten Voraussetzungen erfüllt. Dem Modell liegt eine Stichprobe bestehend aus *N* = 2443 geimpften und *N* = 2338 ungeimpften Pfleger:innen zugrunde. Für das Pflegepersonal hingen alle untersuchten psychologischen Determinanten signifikant mit dem Impfverhalten zusammen. Alleine das Geschlecht war nicht signifikant mit dem Impfverhalten der Pflegenden assoziiert. Auch hier zeigen die Odds Ratios von *Collective Responsibility* sowie dem Vertrauen in die Sicherheit der Impfung (*Confidence*) die stärkste Assoziation mit dem Impfverhalten. Die Chance, geimpft zu sein, erhöhte sich um den Faktor 1,94, je mehr das Pflegepersonal zugestimmt hat, dass die Impfung eine Maßnahme zum Schutz von Menschen mit schwachem Immunsystem ist. Mit jedem Skalenpunkt zunehmender Zustimmung, Vertrauen in die Sicherheit der Impfung zu haben, erhöhte sich die Chance des Pflegepersonals, geimpft zu sein, um den Faktor 1,9. Auch das Vertrauen in die Wirksamkeit der Impfung (*Confidence*) hing bei dem Pflegepersonal signifikant mit dem Impfverhalten zusammen, jedoch impliziert das Odds Ratio eine vergleichsweise schwache positive Assoziation (OR = 1,18; 95 % KI: 1,04–1,34). Negativ mit dem Impfverhalten assoziiert waren – wie auch bei der Ärzteschaft – *Constraints, Complacency* und *Calculation *(Abb. 2).

**Abb. 3:**
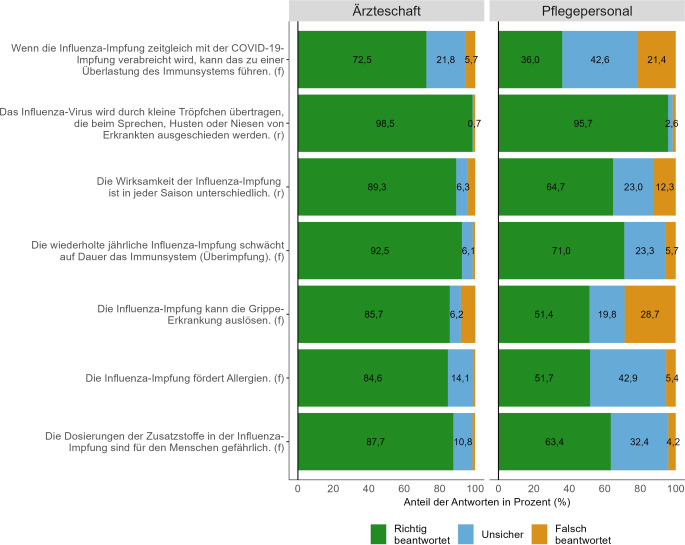
Antworten der Wissensitems von der Ärzteschaft und dem Pflegepersonal (f = falsche Aussage, r = richtige Aussage). *Quelle*: eigene Abbildung

Das Wissen zur Influenza-Impfung und zur Influenza-Erkrankung wurde für die Ärzteschaft und das Pflegepersonal getrennt untersucht. Die Mittelwerte der Wissensscores (Spannweite 0–7) unterscheiden sich signifikant zwischen der Ärzteschaft (*M* = 6,1) und dem Pflegepersonal (*M* = 4,3; *t*(7211) = 42,87; *p* < 0,001). Abb. 3 zeigt die Wissensitems und die Antworten der Ärzteschaft und des Pflegepersonals als prozentuale Anteile. Im Durchschnitt wurden von den Ärzt:innen 87,2 % der 7 Wissensitems richtig beantwortet, 3,4 % wurden falsch beantwortet und weitere 9,5 % wurden mit Unsicherheit beantwortet. Bei den durch Pfleger:innen beantworteten Wissensitems lag der Anteil richtiger Antworten bei 62,0 %, der Anteil falscher Antworten bei 11,4 % und der Anteil unsicherer Antworten bei 26,6 %.

Der Fakt zur Virusübertragung durch eine Tröpfcheninfektion wurde sowohl von der Mehrheit der Ärzteschaft (98,5 %) als auch des Pflegepersonals (95,7 %) korrekt beantwortet. 42,9 % des befragten Pflegepersonals und 14,1 % der Ärzteschaft beantwortete die Falschaussage, die Impfung fördere Allergien, mit „unsicher“. Der höchste Anteil falscher Antworten des Pflegepersonals liegt mit 28,7 % bei der Falschaussage, die Impfung könne die Grippeerkrankung selbst auslösen. Auch von den Ärzt:innen fiel der größte Anteil falscher Antworten auf dieses Item (8,2 %). Die falsche Aussage, dass eine zeitgleiche Gabe der Influenza- und COVID-19-Impfung zu einer Überlastung des Immunsystems führen könne, erkennen 72,5 % der Ärzteschaft bzw. 36,0 % des Pflegepersonals als Falschaussage. Damit wurde dieses Wissensitem von den wenigsten Befragten richtig beantwortet.

## Diskussion

Die Befragung des Klinikpersonals ergab für die Saison 2022/2023 große Unterschiede im Impfverhalten zwischen der Ärzteschaft und dem Pflegepersonal. Die Impfentscheidung beider Berufsgruppen hängt stark mit dem Vertrauen in die Sicherheit der Impfung (*Confidence*), dem gemeinschaftlichen Gedanken von Impfen (*Collective Responsibility*) und Zugangsbarrieren (*Constraints*) zusammen. Unsicherheiten und Falschwissen zur Impfung und der Influenza-Erkrankung betreffen in beiden Berufsgruppen vor allem Sicherheitsfragen zur Influenza-Impfung. Bei dem Pflegepersonal kommen Unsicherheiten und Falschwissen zur Influenza-Impfung und zur Erkrankung deutlich häufiger vor als bei der Ärzteschaft. Im Mittel können Angehörige der Ärzteschaft 2 Wissensfragen mehr richtig beantworten als dem Pflegepersonal zugehörige Mitarbeitende.

Ein Vergleich der Impfquoten für die Saison 2022/2023 zeigte, dass Männer sich häufiger impfen ließen als Frauen und die Inanspruchnahme mit zunehmender Altersgruppe stieg. Der Geschlechterunterschied könnte mit der Geschlechterverteilung in den Berufsgruppen zusammenhängen. Die Impfquotenunterschiede zwischen den Berufsgruppen werden im Folgenden diskutiert. Die gefundenen Impfquotenunterschiede nach Alter bestehen auch in der Indikationsgruppe von Personen mit impfrelevanten Grunderkrankungen [25] und lassen sich durch ein im Alter erhöhtes Risiko für eine schwere Influenza-Erkrankung erklären [26, 27]. Außerdem konnten Berufsgruppenunterschiede festgestellt werden: Von der Ärzteschaft waren deutlich mehr Personen geimpft als von dem Pflegepersonal; die Impfquoten unterscheiden sich um 29,6 Prozentpunkte. Dieser Unterschied wurde bereits in früheren Studien zum Impfverhalten von medizinischem Personal vor der COVID-19-Pandemie für Deutschland [15, 17, 22, 28] und für andere europäische Länder berichtet [29–32]. Schon in der Saison 2006/2007 wurde ein Impfquotenunterschied von 21,4 Prozentpunkten zwischen den beiden Berufsgruppen in Deutschland festgestellt [28]. In der Saison 2019/2020 war die Impfquote der Pfleger:innen im Vergleich zu den Ärzt:innen in Deutschland um 31,6 Prozentpunkte geringer [15] und damit vergleichbar mit Ergebnissen aus anderen europäischen Ländern wie Frankreich (31,1 %-Punkte; [29]), Griechenland (32,3 %-Punkte; [30]) und Polen (36,4 %-Punkte; [31]). Irland weist mit 18,3 Prozentpunkten einen geringeren Unterschied zwischen Ärzt:innen und Pfleger:innen in der Saison 2019/2020 auf [32].

Mit dem 5C-Modell von Betsch et al. (2018) können psychologische Determinanten von Impfverhalten identifiziert werden [23]. Es wurde für die Stichprobe der Ärzteschaft und Pfleger:innen getrennt angewendet, um mögliche Unterschiede nach Berufsgruppen zu erkennen und insbesondere für das Pflegepersonal Ansatzpunkte zur Steigerung der Impfinanspruchnahme zu identifizieren.

Das Verständnis der eigenen Impfung als Schutzmaßnahme für vulnerable Personen hing in der Ärzteschaft und unter dem Pflegepersonal stark positiv mit dem Impfverhalten zusammen. In früheren Studien wurde bereits gezeigt, dass bei medizinischem Personal die Wahrnehmung der Influenza-Impfung als Maßnahme zum Schutz anderer eine relevante Impfmotivation war [22, 33, 34]. Die Ansprache von *Collective Responsibility* könnte also auch bei Ärzt:innen und Pfleger:innen in Deutschland zu einer Steigerung der Impfquoten führen. Der Nutzen der Impfung, auch andere durch die eigene Impfung zu schützen, ist im Sinne des Berufsethos von medizinischem Personal, vulnerable Menschen zu schützen. Diese Motivation kann genutzt werden.

Weiterhin stark positiv mit dem Impfverhalten assoziiert war *Confidence* als Vertrauen in die Sicherheit der Impfung. Vertrauen konnte als wesentliche Determinante von Influenza-Impfverhalten bei medizinischem Personal bereits vielfach bestätigt werden [33, 35]. Demnach führten fehlendes Vertrauen in die Sicherheit und Wirksamkeit der Impfung zur Entscheidung gegen die Impfung. In dieser Untersuchung konnte ein Zusammenhang zwischen Impfverhalten und dem Vertrauen in die Wirksamkeit bei Ärzt:innen nicht bestätigt werden und bei Pfleger:innen war dieser sehr schwach ausgeprägt. Eine mögliche Erklärung liefern die vorliegenden Studienergebnisse zum Wissen: Fast 90 % der Ärzt:innen und 65 % der Pflegenden wissen um die jährlich schwankende Wirksamkeit der Influenza-Impfung Bescheid. Das Wissen zu Impfungen im Allgemeinen und das allgemeine Vertrauen in Impfungen hängen nachweislich zusammen [23].

*Constraints* hingen in beiden Berufsgruppen stark negativ mit dem Impfverhalten zusammen. Demnach führte verstärkter wahrgenommener Alltagsstress dazu, dass Ärzt:innen und Pfleger:innen die jährliche Influenza-Impfung eher nicht wahrnahmen. Neufeind et al. (2021) fanden diese Barriere schon früher bei Ärzt:innen in deutschen Kliniken, jedoch weniger ausgeprägt beim Pflegepersonal. Die Autor:innen fanden außerdem, dass sich ein Impfangebot vor Ort in der Klinik positiv auf die Inanspruchnahme der Influenza-Impfung auswirken kann [22]. Ein systematisches Review stützt diese These mit der Erkenntnis, dass Interventionen zur Erleichterung des Zugangs zur Impfung bzw. zur komfortableren Gestaltung sich als wirksam für die Steigerung von Impfquoten erwiesen haben [18].

In beiden Berufsgruppen wurde außerdem eine negative Assoziation von *Complacency *mit dem Impfverhalten identifiziert. Das heißt, Ärzt:innen und Pfleger:innen, die die Grippeerkrankung als nicht schwer genug einschätzten, ließen sich eher nicht impfen. Eine geringe Risikowahrnehmung der Erkrankung konnte bereits vor der Pandemie als hinderlicher Faktor bei Ärzt:innen und Pfleger:innen in Deutschland identifiziert werden [22]. Auch andere Untersuchungen erkannten eine geringe Risikowahrnehmung als Barriere bei der Influenza-Impfung für medizinisches Personal [36, 37]. Die Risikowahrnehmung spielt bei der Impfentscheidung jedoch eine vergleichsweise geringere Rolle als andere Faktoren [34]. Das zeigen auch die Ergebnisse der vorliegenden Studie.

Das Wissen kann neben den psychologischen Determinanten Einfluss auf die Impfentscheidung nehmen. Es wurden wesentliche Wissenslücken zur Influenza-Impfung und der Erkrankung identifiziert, insbesondere unter dem Pflegepersonal. Gleichzeitig hängen das Vertrauen in die Impfung (*Confidence*) und die Risikowahrnehmung (*Complacency*) mit dem Wissen zusammen [23]. Die Ergebnisse zeigen, dass fehlendes Vertrauen in die Impfstoffsicherheit und eine geringe Risikowahrnehmung eher zur Entscheidung gegen die Impfung führten. Unterdessen wurden teils große Unsicherheit und wesentliches Falschwissen insbesondere bei Fragen zur Sicherheit der Impfung nachgewiesen. Der Großteil des Pflegepersonals und mehr als ein Viertel der Ärzteschaft wussten nicht um die sichere Koadministration der Influenza- und COVID-19-Impfstoffe. Zudem waren sich fast ein Fünftel der Ärzt:innen und über 40 % der Pfleger:innen unsicher, ob die Influenza-Impfung Allergien auslösen könne (falsch). Fast ebenso viel Unsicherheit bestand zur falschen Aussage, dass Zusatzstoffe in der Influenza-Impfung in gefährlichen Mengen dosiert seien. Wissenslücken zur Influenza-Impfung wurden bei medizinischem Personal auch in anderen Studien bestätigt, wobei insbesondere die Falschaussage, die Impfung könne die Erkrankung auslösen, als gängiger Irrglaube identifiziert wurde [33, 35]. Eine mögliche Erklärung für diese beständige Falschinformation könnte die Tatsache sein, dass sich Impfreaktionen zur Influenza-Impfung wie sehr schwache Grippesymptome anfühlen können. Interventionen mit dem Ziel, das Wissen rund um die Impfung zu verbessern, bieten die Chance, auch die Inanspruchnahme positiv zu beeinflussen [18]. Dabei sollten insbesondere Sicherheitsfragen adressiert werden, um die hier identifizierten Wissenslücken zu schließen. Studien beweisen immer wieder, dass der Ärzteschaft bei Fragen zur Impfung das höchste Vertrauen entgegengebracht wird [38]. Das Einbinden der Betriebsmediziner:innen der Krankenhäuser kann demnach sinnvoll sein, z. B. indem sie in ihrer Aufklärungsarbeit gestärkt und unterstützt werden, wenn es darum geht, Fragen zur Sicherheit zu beantworten und mit gängigen Vorbehalten umzugehen. Eine systematische Übersichtsarbeit hat gezeigt, dass die effektivsten Interventionen zur Steigerung von Impfquoten aus mehreren Komponenten bestanden und dass eine Ausrichtung auf definierte Zielgruppen sowie auf definierte Bedarfe essenziell für das Gelingen solcher Interventionen war [18].

Bei den vorliegenden Ergebnissen sollten Limitationen berücksichtigt werden, die aus dem Studiendesign resultieren. Die Befragung wurde einem multizentrischen Studiendesign folgend durch das Robert Koch-Institut durchgeführt. Demnach ist ein Selektionsbias auf Ebene der Kliniken und auf Ebene der Mitarbeitenden wahrscheinlich. Kliniken und Mitarbeitende, die eine positive Einstellung zum Robert Koch-Institut, Gesundheitsthemen und der Influenza-Impfung im Speziellen haben, nehmen möglicherweise eher teil. Zusätzlich können es eben diese Kliniken sein, denen die Relevanz der Influenza-Impfung für ihre Mitarbeiter:innen bewusst ist und die entsprechende Maßnahmen bereits ergriffen haben. Als Resultat kann von einer Überschätzung der Impfquote ausgegangen werden. Die Ergebnisse der Unterschieds- sowie Zusammenhangsanalysen sind von dieser potenziellen Verzerrung nicht betroffen, da nicht anzunehmen ist, dass ein möglicher Selektionsbias in den betrachteten Zielgruppen unterschiedlich ausfällt. Insgesamt wurde eine große deutschlandweite Stichprobe befragt, die jedoch nur einen kleinen Teil aller Klinikmitarbeitenden in Deutschland abdeckt: Mit insgesamt 115 Kliniken konnten 6,1 % aller Kliniken in Deutschland erreicht werden. Es nahmen 8,4 % aller Mitarbeitenden in den Kliniken teil, was in Summe 15.312 Teilnehmende bedeutet. Generell gilt, dass eine sehr hohe Teilnahmequote die Grundgesamtheit (hier Klinikpersonal) besonders gut abbilden und damit das Risiko eines Selektionsbias deutlich reduzieren kann. Es war nicht möglich, die Stichprobe zu gewichten, da in dem Bericht der Krankenhausstatistik notwendige Indikatoren für eine Gewichtung fehlen [24]. Somit ist eine Angleichung der Stichprobe an die Grundgesamtheit von Klinikmitarbeitenden in Deutschland nach den Merkmalen Region, Beruf, Alter und Geschlecht nicht möglich. OKaPII ist eine Querschnittbefragung, sodass keine Aussagen zur Kausalität möglich sind. Gleichwohl bieten die berichteten Ergebnisse einen relevanten Einblick in das Impfverhalten von Ärzt:innen und Pfleger:innen in Deutschland. Es werden behaviorale Faktoren berücksichtigt, die ein tiefergehendes Verständnis – über die reine Impfquote hinaus – ermöglichen. OKaPII ist das einzige Monitoring-Instrument dieser Art in Deutschland. Eine wesentliche Stärke der Studie besteht in dem klinikspezifischen Ergebnisbericht, den alle teilnehmenden Kliniken erhalten haben. Damit befähigt die Studie die relevanten Akteur:innen, selbst evidenzbasierte Maßnahmen zur Steigerung der Influenza-Impfquote für die Klinikmitarbeitenden zu ergreifen.

## Fazit

In der Influenza-Saison 2022/2023 bestanden relevante Impflücken bei Klinikpersonal in Deutschland. Besonders gering war – wie auch in vorherigen Jahren – die Impfquote des Pflegepersonals im Vergleich zu anderen Berufsgruppen in deutschen Kliniken. Die vorliegenden Erkenntnisse weisen darauf hin, dass insbesondere die Stärkung des Vertrauens in die Sicherheit der Impfung, die Vermittlung von Impfen als gemeinschaftliche Maßnahme zum Schutz anderer und der Abbau von zeitlichen Barrieren bei der Inanspruchnahme wichtige Ansatzpunkte sein können, um die Impfquoten der Ärzteschaft und des Pflegepersonals zu steigern. In beiden Berufsgruppen betreffen Unsicherheiten und Falschwissen vor allem Sicherheitsfragen zur Influenza-Impfung. Maßnahmen, die auf diese Zielgruppen und Bedarfe ausgerichtet sind, können die Impfquote steigern und damit das Potenzial der Influenza-Impfung im klinischen Setting besser nutzbar machen. Eine Multikomponentenintervention erhöht zusätzlich die Chance, dass die Influenza-Impfung vermehrt wahrgenommen wird. Dabei erscheint es auch sinnvoll, vertrauenswürdige Personen aus dem jeweiligen Setting einzubeziehen, die aufklären und gleichzeitig das Vertrauen in die Impfung stärken können.

## Supplementary Information

Das ergänzende Material enthält eine Auflistung der Konstrukte mit Items und Antwortkategorien des OKaPII-Fragebogens sowie eine Beschreibung der Klinikstichprobe.

## Footnotes

1 https://osf.io/4ykfd/?view_only=6b3a27cd63094bb6831ba02484024d93.
