# Supplementary material for: Understanding vaccination behavior of healthcare workers in German hospitals—results from the OKaPII study
Source: Bundesgesundheitsblatt Gesundheitsforschung Gesundheitsschutz. 2024 Dec 3;68(1):79–87. [Article in German] doi: 10.1007/s00103-024-03982-7 (PMC11732876; doi:10.1007/s00103-024-03982-7)
Supplement: Supplementary file 1 — Das ergänzende Material enthält eine Auflistung der Konstrukte mit Items und Antwortkategorien des OKaPII-Fragebogens sowie eine Beschreibung der Klinikstichprobe. [file 103_2024_3982_MOESM1_ESM.pdf]

Onlinematerial zu „Impfverhalten von Klinikpersonal verstehen – Ergebnisse der OKaPII-Studie 2023“ von E. Wulkotte und N. Schmid-Küpke

Tabelle A1. *Konstrukte mit Items und Antwortkategorien des OKaPII-Fragebogens (Reihenfolge entspricht der Abfrage).*

Konstrukt	Item	Antwortkategorien	Anzahl der Items, Format, Hinweise
Alter	Wie alt sind Sie? Ich bin…	Auswahl: 16-99 Jahre	1 Item<br><br>Bildung von Kategorien:<br>18 – 29<br>30 – 39<br>40 – 49<br>50 – 59<br>≥ 60<br><br>Fragebogen wurde beendet, wenn Alter ≤ 17 Jahre
Geschlecht	Welche Bezeichnung trifft auf Sie am ehesten zu?	Männlich<br>Weiblich<br>Divers	1 Item
Klinik	An welchem Krankenhaus sind Sie beschäftigt? Wählen Sie zuerst das entsprechende Bundesland und dann Ihr Krankenhaus aus.	Auswahl: Bundesland<br>Baden-Württemberg<br>Bayern<br>Berlin<br>Brandenburg<br>Bremen<br>Hamburg<br>Hessen<br>Mecklenburg-Vorpommern<br>Niedersachsen	2 Items

**Konstrukt**	**Item**	**Antwortkategorien**	**Anzahl der Items, Format, Hinweise**
		Nordrhein-Westfalen<br>Rheinland-Pfalz<br>Saarland<br>Sachsen<br>Sachsen-Anhalt<br>Schleswig-Holstein<br>Thüringen<br><br>Auswahl: Kliniken<br>Liste der teilnehmenden Kliniken	
Beruf	Welcher Berufsgruppe gehören Sie an?	Ärztlicher Dienst<br>Pflegepersonal<br>Medizinisch-technisches Personal (z.B. Radiologie-technische Assistenz, Ergometrie, usw.)<br>Therapeutische Berufe (z.B. Physiotherapie, Logopädie, usw.)<br>Laborpersonal<br>Küchenpersonal oder Hauswirtschaft<br>Reinigungspersonal<br>Verwaltung<br>Sonstige	1 Item
Impfstatus	Haben Sie sich in der letzten Saison, d.h. zwischen September 2022 bis einschließlich März 2023, gegen Grippe impfen lassen?	Ja<br>Nein	1 Item

Konstrukt	Item	Antwortkategorien	Anzahl der Items, Format, Hinweise
5C psychologische Determinanten von Impfverhalten	Ich habe vollstes Vertrauen in die Sicherheit der Grippeschutzimpfung. (*Confidence*)<br><br>Die Grippeschutzimpfung ist wirksam. (*Confidence*)<br><br>Die Grippe ist nicht so schlimm, dass ich mich gegen sie impfen lassen müsste. (*Complacency*)<br><br>Alltagsstress hält mich davon ab, mich gegen Grippe impfen zu lassen. (*Constraints*)<br><br>Wenn ich darüber nachdenke, mich gegen Grippe impfen zu lassen, wäge ich sorgfältig Nutzen und Risiken ab. (*Calculation*)<br><br>Ich lasse mich gegen Grippe impfen, weil ich so auch Menschen mit einem schwachen Immunsystem schützen kann. (*Collective Responsibility*)	1 = Stimme überhaupt nicht zu<br>2<br>3<br>4<br>5 = Stimme voll und ganz zu	6 Items<br><br>Referenz: Betsch et al., 2018

Konstrukt	Item	Antwortkategorien	Anzahl der Items, Format, Hinweise
Wissen	Die Dosierungen der Zusatzstoffe in der Grippeschutzimpfung sind für den Menschen gefährlich. (f)<br><br>Die Grippeschutzimpfung fördert Allergien. (f)<br><br>Die Grippeschutzimpfung kann die Grippeerkrankung auslösen. (f)<br><br>Die wiederholte jährliche Grippeschutzimpfung schwächt auf Dauer das Immunsystem (Überimpfung). (f)<br><br>Die Wirksamkeit der Grippeschutzimpfungen ist in jeder Saison unterschiedlich. (r)<br><br>Das Influenza-Virus wird durch kleine Tröpfchen übertragen, die beim Sprechen, Husten oder Niesen von Erkrankten ausgeschieden werden. (r)<br><br>Wenn die Grippeschutzimpfung zeitgleich mit der COVID-19-Impfung verabreicht wird, kann das zu einer Überlastung des Immunsystems führen. (f)	Ja, stimmt<br>Nein, stimmt nicht<br>Weiß nicht	7 Items<br><br>(r) – richtige Aussage<br>(f) – falsche Aussage<br>Im Fragebogen wurde nur die Aussage abgebildet<br><br>Bildung einer Wissensskala: Richtige Antworten mit 1 codiert, falsche und „weiß nicht“ Antworten mit 0 codiert

Tabelle A2. *Beschreibung der Klinikstichprobe.*

	**Absolute Häufigkeit (N)**	**Relative Häufigkeit (%)**
**Träger**		
Öffentlich	54	47,0
Freigemeinnützig	45	39,1
Privat	16	13,9
**Versorgungsstufe oder Kategorie**		
Grund- oder Regelversorgung	47	40,9
Schwerpunkt-Versorgung	29	25,2
Andere	14	12,2
Maximalversorgung	13	11,3
Universitätsklinikum	7	6,1
Rehaklinikum	3	2,6
Unsicher	2	1,7
	**Mittelwert**	**Standard-abweichung**
**Betten**		
	493,6	413,0
**Beschäftigte**		
	1606,2	2162,8
